# On the Principle of Synchronization

**DOI:** 10.3390/e20100741

**Published:** 2018-09-28

**Authors:** Andreas Schlatter

**Affiliations:** Burghaldeweg 2F, 5024 Küttigen, Switzerland; schlatter.a@bluewin.ch; Tel.: +41-(0)62-827-00-16

**Keywords:** entropy, thermal clocks, quantum measurement, gravity

## Abstract

We construct a type of thermal quantum-clocks and show that various interesting relations between energy, entropy and geometry in space–time directly follow by partially synchronizing them in the sense of making them march in step with photon clocks.

## 1. Introduction

A quantum clock is a quantum system whose dynamics are coupled to another system and, hence, the former defines a time-scale for the latter [1,2]. In this paper, we will use, as a base, the idea of thermal quantum clocks, introduced in [2,3], which are universal in the sense that they define the duration of a general quantum-measurement. We show that the partial synchronization of such clocks, in the sense of demanding them to march in step, allows, in appropriate physical situations, the straightforward derivation of some key relations between energy, entropy, and geometry in space–time. Among them are the Davies–Unruh effect [4,5], the Ehrenfest–Tolman effect [6], the Hawking temperature of black holes [7], the Bekenstein bound, a holographic bound [8,9], and related relations in de-Sitter vacuum. Finally, we show that by covariant extension of a particular synchronization, we are led to Einstein’s equations. Each of the relations can be derived within its own theoretical framework, sometimes with considerable effort. With the synchronization principle, we present an elementary way of deriving them all out of a single principle. This is re-approved evidence, that measuring time by means of electromagnetic energy [10,11,12] plays a key role in the physics of space–time.

## 2. Thermal Clocks 

Consider a quantum system in a pure state $|\Pi \rangle =\sum_{i\epsilon I}c_{i}|\Pi_{i}\rangle$, where $c_{i}\epsilon \mathbb{C}$ and ${\left\{|\Pi_{i}\rangle \right\}}_{i\epsilon I}$ is an eigenbasis of some observable $\hat{O}$. A measurement leads to the symbolic sequence

(1) $$|\Pi \rangle \to |\Pi \rangle \langle \Pi |\to |\Pi_{i_{0}}\rangle ,i_{0}\epsilon I.$$

The probability of outcome $|\Pi_{i_{0}}\rangle$ is ${\left|c_{i_{0}}\right|}^{2}$. After an increase in entropy in the first part, there is an entropy decrease in the second part of sequence (1), when the outer observer “learns” the result. To harmonize with the second law and with the experience of an inner observer, there must be an entropy dissipation to the environment of a minimal amount of $S=-tr\left(\varrho \log_{2}\varrho \right)=-\sum_{i\epsilon I}{\left|c_{i}\right|}^{2}\log_{2}{\left|c_{i}\right|}^{2}$, where ϱ=|Π〉〈Π| [13,14] (the dissipation is the result of erasure of a former state). If the environment is in thermal equilibrium at temperature T, then this leads to an average energy-flow into the environment of $\bar{E}=Sk_{B}T$, where $k_{B}$ is Boltzmann’s constant. The energy is radiation by harmonic oscillators with frequency $h\nu \ll k_{B}T$ and, hence, average energy $E_{\nu }\approx k_{B}T$, where h is Planck’s constant. By a result in [15], the evolution of a quantum system with average energy $\bar{E}$ takes, minimally, a time interval of $\Delta t=\frac{h}{4\left(\bar{E}-E_{0}\right)}$ to flip to an orthogonal state, where $E_{0}$ denotes the energy of the ground state. Hence, the environment $|E_{0}\rangle$ takes (minimally) the time-amount of

(2) $$\Delta t=\frac{h}{4k_{B}TS}$$

to reach an orthogonal state $|E_{1}\rangle$, $\langle E_{0}|E_{1}\rangle =0$. The environment, therefore, serves as an ideal thermal clock, which takes the interval (2) for one “tick” to indicate that the measurement (1) has been completed with certainty [16].

For any time-parameter τϵℝ we can, with the help of (2), define a time-scale by dividing the time-continuum into equal steps by the ratio

(3) $$dr=\frac{d\tau }{\Delta t}=\left(\frac{4k_{B}}{h}TS\right)d\tau .$$

We call two thermal clocks partially synchronized, if they march in step, i.e., for some constant α>0, it holds that

(4) $$dr_{1}=\alpha dr_{2}.$$

## 3. Principle of Synchronization

### 3.1. Tolman–Ehrenfest and Davies–Unruh Effect

Assume there is a static space–time manifold (M,g) and two observers at space–time points, $x_{1}\epsilon M,x_{2}\epsilon M,$ locally at rest; partially synchronize their thermal clocks. In a static metric, the eigen-time interval for an observer, who is locally at rest, is $d\tau =\sqrt{g_{00}}dt$. Condition (4), together with (3), then directly translates into

(5) $$S_{1}T\left(x_{1}\right)\sqrt{g_{00}\left(x_{1}\right)}=\alpha S_{2}T\left(x_{2}\right)\sqrt{g_{00}\left(x_{2}\right)}.$$

If we set $\alpha =\frac{S_{1}}{S_{2}}$, then (5) is the condition for thermal equilibrium in a gravitational field. The position-dependence of temperature is also known as *Tolman–Ehrenfest effect* [6]. Equally, in spirit, we can ask that two thermal clocks in Minkowski space–time are synchronized, where one is in relative motion at constant speed v to the other. With $\gamma =\frac{1}{\sqrt{1-\frac{v^{2}}{c^{2}}}}$, there holds for time in the moving coordinates $dt^{\prime }=\gamma \cdot dt$. Hence, if $T^{\prime }$ denotes the temperature in the moving coordinates, relation (4) translates (with the same α as in (5)) into

(6) $$T^{\prime }dt^{\prime }=T^{\prime }\gamma dt=Tdt.$$

Therefore,

(7) $$T^{\prime }=\left(\sqrt{1-\frac{v^{2}}{c^{2}}}\right)T.$$

Relation (7) corresponds to the original formula for relativistic temperature transformation in [17]. After these introductory examples, we now consider a uniformly accelerated observer in Minkowski space–time, who moves with acceleration κ in x-direction, say. We chose a co-moving coordinate system, which is defined in the wedge, limited by |x|=t, and given by the transformations

(8) x=ϱcosh(κϑ), t=ϱsinh(κϑ),      ϱ≥0, −∞<ϑ<∞.

The eigen-time element is

(9) $$d\tau^{2}=\frac{1}{c^{2}}ds^{2}={\left(\frac{\kappa \varrho }{c^{2}}\right)}^{2}d\vartheta^{2}-\frac{1}{c^{2}}\left(d\rho^{2}+dy^{2}+dz^{2}\right).$$

These are so-called Rindler coordinates [18]. At t=0, the observers are at rest in their respective local inertial frames, and ratio (3) takes the form

(10) $$dr=\frac{4k_{B}}{h}TS\frac{\kappa \varrho }{c^{2}}d\vartheta .$$

A thermal clock in the local inertial frame at $\varrho_{1}=\frac{c^{2}}{\kappa }$ and another one stationary at $\varrho_{2},$ say, are synchronized by (4), if

(11) $$dr_{1}=\alpha dr_{2}.$$

Hence, if $\alpha =\frac{S_{1}}{S_{2}}$, which we from now on assume, if not stated otherwise,

$$dr_{1}=\frac{4}{h}k_{B}T_{\left(\varrho =\frac{c^{2}}{\kappa }\right)}\times d\vartheta =dr_{2}=\frac{4}{h}k_{B}T_{\varrho_{2}}\times \varrho_{2}\times \frac{\kappa }{c^{2}}\times d\vartheta .$$

With $T_{\kappa }\cdot T_{\left(\varrho =\frac{c^{2}}{\kappa }\right)}$, we arrive at

(12) $$v_{\kappa }=\frac{4}{h}k_{B}T_{\kappa }\times \frac{c^{2}}{\kappa }=\frac{4}{h}k_{B}T_{\varrho_{2}}\times \varrho_{2}=const.$$

So far, we have worked with a single acceleration or chart. We can generalize (12) by demanding it to hold for different accelerations $\kappa_{1}\neq \kappa_{2}$, which is the basis for the generalization to non-flat manifolds like in (5). For our purpose, we consider a specific second system, namely, a single oscillator of frequency ν with energy of one photon $E=\frac{3}{2}h\nu$. For its energy above the ground state, we have

(13) $$\Delta E=\frac{3}{2}h\nu -\frac{1}{2}h\nu =h\nu .$$

With  ω=2πν and λ being the wavelength, the acceleration is $\kappa =\lambda \omega^{2}$, and the left-hand side of (12) turns into

(14) $$v_{\omega }=\frac{4}{h}\times \hbar \omega \times \frac{c^{2}}{\lambda \omega^{2}}=\frac{2c^{2}}{\pi \lambda \omega }=\frac{2c^{2}}{2c\pi^{2}}=\frac{c}{\pi^{2}}.$$

$v_{\omega }$ is independent of ω and synchronizing $v_{\kappa }=v_{\omega }$ implies

(15) $$\frac{4}{h}k_{B}T_{\kappa }\times \frac{c}{\kappa }=\frac{1}{\pi^{2}}.$$

From (15), we get for the corresponding temperature $T_{\kappa }$

(16) $$T_{\kappa }=\frac{\hbar \kappa }{2\pi ck_{B}}.$$

Expression (16) is the *Unruh–Davies temperature* [4,5]. From Equation (15), there immediately results a number of well-known temperature expressions, if the acceleration κ is chosen appropriately. In case of a Schwarzschild black hole of mass M, for instance, κ is he surface gravity on the horizon $\kappa =\frac{c^{4}}{4MG}$, where G denotes the gravitational constant, and we get the Hawking temperature

(17) $$T_{\kappa }=\frac{\hbar c^{3}}{8\pi k_{B}MG}.$$

Similar expressions hold for Reissner–Nordström or Kerr black holes, if the corresponding surface accelerations are plugged into (16).

### 3.2. Bekenstein-Type of Bounds on Entropy

Relations (15) and (16) can be used to derive some further interesting results. Let the total energy within a ball of radius R=λ in flat space be $E_{tot}^{R}$, as measured in the local rest frame of a single photon clock. Clearly the total dissipation out of this region for any thermodynamic entropy S must satisfy

(18) $$T_{\frac{c^{2}}{R}}S\leq E_{tot}^{R}.$$

Hence, with (16) for $\kappa =\frac{c^{2}}{R}$, we directly get the Bekenstein-bound [7]

(19) $$S\leq \frac{2\pi k_{B}R}{\hbar c}E_{tot}^{R}.$$

Instead of the photon rest-frame, we chose the local rest-frame of an observer, who is (weakly) gravitationally attracted by a mass M, relatively at rest at R. In the Newtonian limit with potential $\varphi =-\frac{GM}{R},$ there is the local acceleration $g_{R}=\frac{MG}{R^{2}}$, where G is the gravitational constant. In the presence of matter, we have to slightly change the synchronization Equation (15) by adding an additional energy quantum to the photon-clock in vacuum (hence we actually synchronize with a two-photon clock, one energy quantum for space and one for matter), leading to

(20) $$\frac{4}{h}k_{B}T_{g_{R}}\frac{c}{g_{R}}=\frac{2}{\pi^{2}}.$$

Like in (18), there must hold

(21) $$T_{g_{R}}S\leq E_{tot}^{R}.$$

With the total energy $E_{tot}^{R}=Mc^{2}$, we get from (20)

(22) $$\frac{\hbar MG}{\pi k_{B}cR^{2}}S\leq Mc^{2},$$

and, finally, with the definition of the Planck length $l_{P}=\sqrt{\frac{G\hbar }{c^{3}}}$ and $A_{R}=4\pi R^{2}$, denoting the surface of the sphere with radius R, there directly results the area law [8]

(23) $$S\leq \frac{k_{B}A_{R}}{4l_{P}^{2}}.$$

Estimate (23) remains true, and can be derived from (16) for the surface area of a Schwarzschild black hole, if we set $\kappa =\frac{c^{4}}{4MG}=\frac{2GM}{R_{S}^{2}}$ with $R_{S}=\frac{2GM}{c^{2}}$ denoting the Schwarzschild radius.

### 3.3. De-Sitter Vacuum 

We now consider de-Sitter space–time, representing the vacuum with energy density ${\bar{e}}_{vac}=\frac{c^{4}}{8\pi G}\Lambda$, where Λ denotes the cosmological constant. It is well known that this space–time has a horizon $R_{\infty }$ with surface-acceleration $a_{\infty }$ given by the Hubble constant $H_{0}$. There holds

(24) $$a_{\infty }=cH_{0}=c^{2}\sqrt{\frac{\Lambda }{3}}=\frac{c^{2}}{R_{\infty }}.$$

We have, in analogy to (18) for any thermodynamic entropy S within the region bounded by the horizon,

(25) $$T_{a_{\infty }}S\leq E_{tot}^{R_{\infty }}=\frac{c^{4}}{8\pi G}\Lambda \times vol\left(B_{R_{\infty }}\right).$$

In the static de-Sitter metric (the line-element is $ds^{2}=\left(1-\frac{r^{2}}{R_{\infty }^{2}}\right)dt^{2}-{\left(1-\frac{r^{2}}{R_{\infty }^{2}}\right)}^{-1}dr^{2}-r^{2}d\Omega^{2}$)

(26) vol(BR∞)=∫0R∞4πr2(1−r2R∞2)12dr=π2R∞3.

Hence, by help of relation (24)

(27) $$T_{a_{\infty }}S\leq \frac{3\pi c^{4}R_{\infty }}{8G}.$$

By (16), we arrive at

(28) $$S\leq \frac{3k_{B}\pi^{2}R_{\infty }{}^{2}c^{3}}{4G\hbar },$$

and, finally, with the Planck length $l_{P}=\sqrt{\frac{G\hbar }{c^{3}}}$, we get an area law

(29) $$S\leq \frac{3\pi }{4}\times \frac{k_{B}A_{R_{\infty }}}{4l_{P}^{2}}.$$

For radii $R<R_{\infty }$, however, (26) does not, a priori, reduce to a simple expression. An interesting question is, what the estimate looks like far away from the horizon in the visible universe, i.e., for a ball of radius $R\ll R_{\infty }$. Here, the volume of the ball is almost Euclidean. By Hubble’s law, we have for the surface acceleration $a_{R}=\frac{R}{R_{\infty }}a_{\infty },$ and we get, in analogy to (25) and (27),

(30) $$T_{a_{R}}S\leq \frac{c^{4}}{8\pi G}\Lambda \times vol\left(B_{R}\right)=\frac{c^{4}}{8\pi G}\Lambda \times \frac{4}{3}\pi R^{3}=\frac{c^{4}R^{3}}{2R_{\infty }^{2}G}.$$

In terms of $a_{\infty }$,

$$S\times a_{\infty }\leq \frac{\pi k_{B}c^{5}R^{2}}{R_{\infty }G\hbar }.$$

Finally, again by (24), we get the traditional area law

(31) $$S\leq \frac{k_{B}A_{R}}{4l_{P}^{2}}.$$

It is interesting that the static de-Sitter coordinate frame represents the perspective of a non-co-moving observer, for whom there is a velocity of the “river of space” [19]. Yet, the metric looks entirely static. One can indeed ask how, in a vacuum, one should differentiate between $a_{\infty }$ and $a_{R}$ in the absence of anything to compare. There is the ansatz to attribute the scale factor $\frac{R}{R_{\infty }}$ to the entropy instead, and define $S_{R}\cdot \frac{R}{R_{\infty }}S$ [20]. In Equation (30), we would then start with the expression $T_{a_{\infty }}S_{R}$. This ansatz can be useful, if we are in the Schwarzschild–de-Sitter space–time, which is defined by a gravitating mass M and the expanding vacuum, in combination (the line-element is $ds^{2}=\left(1-\frac{r^{2}}{R_{\infty }^{2}}-\frac{2MG}{r}\right)dt^{2}-{\left(1-\frac{r^{2}}{R_{\infty }^{2}}-\frac{2MG}{r}\right)}^{-1}dr^{2}-r^{2}d\Omega^{2}$). For consistency’s sake, the thermal clocks of two observers, one locally accelerated by $g_{R}=\frac{MG}{R^{2}}$ and the other by $b_{R}$, also taking into account the vacuum expansion, must then march in step (5), i.e., with α=1

(32) $$T_{g_{R}}Sd\tau =T_{b_{R}}S_{R}d\tau .$$

Therefore, with Hubble’s law and $a_{0}=\frac{a_{\infty }}{2}$,

(33) $$\frac{b_{R}^{2}}{a_{0}}=g_{R},$$

which leads to

(34) $$b_{R}=\frac{\sqrt{GMa_{0}}}{R}.$$

Expression (34) is the empirically quite well-tested MOND (Modified Newtonian Dynamics) acceleration [20,21]. It can be shown that the effect of the vacuum expansion will only be relevant for distances $R>R_{0}=\sqrt{\frac{GM}{a_{0}}}$, where (29) does not hold, and where the vacuum acceleration is bigger than $a_{0}$ [22]. From this perspective, the correction (34) to the gravitation law is ultimately due to Λ.

We have seen, so far, that the synchronization principle is a very powerful tool to offer a simple derivation of some key relations in space–time physics. We now want to show that the Einstein equations can also be derived from this principle.

### 3.4. Einstein Equations

We want to come back to the local situation of weak gravitational attraction and use Equation (20).

(35) $$\frac{4}{h}k_{B}T_{g_{R}}\frac{c}{g_{R}}=\frac{2}{\pi^{2}}$$

With $E=Mc^{2}$, we write

(36) $$k_{B}T_{g_{R}}A_{R}=4l_{P}^{2}Mc^{2}=4l_{P}^{2}E.$$

By using (14), we arrive at

(37) $$g_{R}A_{R}=\frac{8\pi G}{c^{4}}E.$$

Equation (37), which holds on a hypersurface in the Newtonian limit, can be covariantly generalized, if we assume a static (or conformal) static metric. We sketch the derivation in, e.g., [23]. We consider an asymptotically flat, static background with global time-like Killing vector field $\xi^{a}$. The generalization of Newton’s potential φ can be defined by

(38) $$\varphi =\frac{1}{2}log\left(-\xi^{a}\xi_{a}\right).$$

The exponential $e^{\varphi }$ is the red-shift factor, which defines a foliation of space–time in space-like surfaces S of constant red-shift. In this set-up, a particle on the corresponding Killing world-line will have a four-acceleration perpendicular to S given by

(39) $$a^{b}=-\nabla^{b}\varphi .$$

The left-hand side of (37) formally turns into the more general expression

(40) $$g_{R}A_{R}\to \overset{}{\underset{S}{\int }}e^{\varphi }\nabla \varphi \cdot dA.$$

Relation (40) is (modulo constants) exactly the expression for the Komar-mass M, [24], and we indeed get, by accounting for the constants, the equivalent equation to (37):

(41) $$\frac{1}{2}\overset{}{\underset{S}{\int }}e^{\varphi }\nabla \varphi \cdot dA=\frac{4\pi G}{c^{2}}\cdot M=\frac{4\pi G}{c^{4}}E.$$

Re-expressed in terms of the Killing vector $\xi^{a}$, there holds

(42) $$\overset{}{\underset{S}{\int }}dx^{a}\wedge dx^{b}\epsilon_{abcd}\nabla^{c}\xi^{d}=\frac{8\pi G}{c^{4}}E.$$

By Stokes theorem and the identity $\nabla^{a}\nabla_{a}\xi^{b}=-R_{a}^{b}\xi^{a}$ (42) turns into

(43) $$\overset{}{\underset{\Sigma }{\int }}R_{ab}n^{a}\xi^{b}dV=\frac{8\pi G}{c^{4}}E,$$

where Σ is a volume bounded by S. Since by (43), the Ricci tensor $R_{ab}$ equals zero in a massless region, relation (43) holds for any boundary surface S of Σ, as long as Σ comprises all the matter. By writing the right-hand side as an appropriate integral over components (the energy field should be source free) of the energy-stress tensor $T_{ab}$, we get

(44) $$\overset{}{\underset{\Sigma }{\int }}R_{ab}n^{a}\xi^{b}dV=\frac{8\pi G}{c^{4}}\overset{}{\underset{\Sigma }{\int }}\left(T_{ab}-\frac{1}{2}Tg_{ab}\right)n^{a}\xi^{b}dV.$$

Finally, by considering an infinitesimally small region and imposing that, if matter m crosses the screen, then the Komar-integral changes by that amount, and (44) can be shown to hold for all (approximate) Killing vectors $\xi^{a}$ and screens S with normal vector $n^{a}$. Therefore,

(45) $$R_{ab}=\frac{8\pi G}{c^{4}}\left(T_{ab}-\frac{1}{2}Tg_{ab}\right).$$

A similar approach was taken in [25] by using null-screens.

## 4. Conclusions

In this paper, we introduced abstract thermal quantum clocks, which are basic in as much as they define a generic duration for any quantum-measurement. Clearly, we are in the tradition to attest to the measurement process, which we do not specify further, additional measurable consequences and hence some real existence. By demanding that these clocks march in step in particular with elementary photon clocks, i.e., by sequencing the “flow of reality” through time intervals proportionate to the ones of oscillating photon-clocks, we were able to easily derive a number of well-established relations between energy, entropy, and geometry in space–time. There has been some doubt whether recording duration by means of the length of world-lines really exhausts the possible operational definitions of time inside a physical object [26]. We have shown that parts of relativistic physics follow from correlating the space–time metric to duration, which originates from electromagnetic oscillation in form of single quanta and thermal radiation. The power of the simple principle turns out to be quite astonishing, and is further proof of the extraordinary importance of electromagnetism for our perception and description of physical time and space.

## References

[1] Peres A. (1980). Measurement of time by quantum clocks. Am. J. Phys..

[2] Schlatter A. (2017). Duration and its relationship to the geometry of Space-Time I. Int. J. Quantum Found..

[3] Schlatter A. (2017). Duration and its relationship to the geometry of Space-Time II. Int. J. Quantum Found..

[4] Unruh W.G. (1976). Notes on black-hole evaporation. Phys. Rev. D.

[5] Davies P.C.W. (1975). Scalar production in Schwarzschild and Rindler metrics. J. Phys. A Math. Gen..

[6] Tolman R.C., Ehrenfest P. (1930). Temperature Equilibrium in a Static Gravitational Field. Phys. Rev..

[7] Hawking S.W. (1975). Particle creation by black holes. Commun. Math. Phys..

[8] Bekenstein J.D. (1981). Universal upper bound on the entropy-to-energy ratio for bounded systems. Phys. Rev. D.

[9] Susskind L. (1995). The world as a hologram. J. Math. Phys..

[10] Einstein A. (1908). Über das Relativitätsprinzip und die aus ihm gezogenen Folgerungen. Jahrb. Radioakt..

[11] Connes A., Rovelli C. (1994). Von Neumann Algebra Automorphisms and Time-Thermodynamics Relation in General Covariant Quantum Theories. Class. Quantum Gravity.

[12] Haggard H., Rovelli C. (2013). Death and resurrection of the zeroth principle of thermodynamics. Phys. Rev. D.

[13] Lubkin E. (1987). Keeping the Entropy of Measurement: Szilard revisited. Int. J. Theor. Phys..

[14] Landauer R. (1961). Irreversibility and Heat Generation in the Computing Process. IBM J. Res. Dev..

[15] Margolus N., Levitin L.B. (1998). The maximum speed of dynamical evolution. Phys. D.

[16] Rovelli C. (1998). “Incerto Tempore, Incertisque Loci”: Can We Compute the Exact Time at Which a Quantum Measurement Happens?. Found. Phys..

[17] Von Mosengeil K. (1907). Theorie der stationären Strahlung in einem bewegten Hohlraum. Ann. Phys..

[18] Rindler W. (1960). Hyperbolic Motion in Curved Space Time. Phys. Rev..

[19] Braeck S., Gron O. (2013). A river model of space. Eur. Phys. J. Plus.

[20] Verlinde E.J. (2017). Emergent Gravity and the Dark Universe. SciPost Phys..

[21] Milgrom M. (2015). MOND-theory. Can. J. Phys..

[22] Schlatter A. (2018). Synchronization of Thermal Clocks and Entropic Corrections of Gravity. Int. J. Quantum Found..

[23] Verlinde E.J. (2011). On the origin of gravity and the laws of Newton. J. High Energy Phys..

[24] Wald R.M. (1984). Asymptotic Flatness. General Relativity.

[25] Jacobson T. (1995). Thermodynamics of Spacetime: The Einstein Equation of State. Phys. Rev. Lett..

[26] Borghi C. (2016). Physical Time and thermal Clocks. Found. Phys..

